# Counselling behavioural interventions for HIV, STI and viral hepatitis among key populations: a systematic review of effectiveness, values and preferences, and cost studies

**DOI:** 10.1002/jia2.26085

**Published:** 2023-05-23

**Authors:** Caitlin E. Kennedy, Ping Teresa Yeh, Annette Verster, Niklas Luhmann, Nabeel Mangadan Konath, Maeve Brito de Mello, Rachel Baggaley, Virginia Macdonald

**Affiliations:** ^1^ Department of International Health Johns Hopkins Bloomberg School of Public Health Baltimore Maryland USA; ^2^ Department of Global HIV Hepatitis and STI Programmes World Health Organization Geneva Switzerland; ^3^ Regional Office for South‐East Asia World Health Organization New Delhi India

**Keywords:** vulnerable populations, counselling, behaviour, HIV, sexually transmitted diseases, [hepatitis, viral, human] systematic review

## Abstract

**Introduction:**

Key populations (sex workers, men who have sex with men, people who inject drugs, people in prisons and other closed settings, and trans and gender diverse individuals) are disproportionately affected by HIV, sexually transmitted infections (STIs) and viral hepatitis (VH). Counselling behavioural interventions are widely used, but their impact on HIV/STI/VH acquisition is unclear.

**Methods:**

To inform World Health Organization guidelines, we conducted a systematic review and meta‐analysis of effectiveness, values and preferences, and cost studies about counselling behavioural interventions with key populations. We searched CINAHL, PsycINFO, PubMed and EMBASE for studies published between January 2010 and December 2022; screened abstracts; and extracted data in duplicate. The effectiveness review included randomized controlled trials (RCTs) with HIV/STI/VH incidence outcomes; secondary review outcomes of unprotected sex, needle/syringe sharing and mortality were captured if studies also included primary review outcomes. We assessed the risk of bias using the Cochrane Collaboration tool, generated pooled risk ratios through random effects meta‐analysis and summarized findings in GRADE evidence profiles. Values and preferences and cost data were summarized descriptively.

**Results:**

We identified nine effectiveness, two values and preferences, and two cost articles. Meta‐analysis of six RCTs showed no statistically significant effect of counselling behavioural interventions on HIV incidence (1280 participants; combined risk ratio [RR]: 0.70, 95% confidence interval [CI]: 0.41–1.20) or STI incidence (3783 participants; RR: 0.99; 95% CI: 0.74–1.31). One RCT with 139 participants showed possible effects on hepatitis C virus incidence. There was no effect on secondary review outcomes of unprotected (condomless) sex (seven RCTs; 1811 participants; RR: 0.82, 95% CI: 0.66–1.02) and needle/syringe sharing (two RCTs; 564 participants; RR 0.72; 95% CI: 0.32–1.63). There was moderate certainty in the lack of effect across outcomes. Two values and preferences studies found that participants liked specific counselling behavioural interventions. Two cost studies found reasonable intervention costs.

**Discussion:**

Evidence was limited and mostly on HIV, but showed no effect of counselling behavioural interventions on HIV/VH/STI incidence among key populations.

**Conclusions:**

While there may be other benefits, the choice to provide counselling behavioural interventions for key populations should be made with an understanding of the potential limitations on incidence outcomes.

## 1 INTRODUCTION

HIV, sexually transmitted infections (STIs) and viral hepatitis (VH) disproportionately affect men who have sex with men, sex workers, people who inject drugs, trans and gender diverse individuals, and people in prisons and other closed settings. These key populations were originally identified as crucial to the HIV response, and they continue to be disproportionately affected by HIV. In 2020, 65% of the 1.5 million new HIV infections globally were among key populations and their sexual partners, and key populations accounted for 93% of new HIV infections outside of sub‐Saharan Africa, and 35% within sub‐Saharan Africa [1]. However, these same populations are also disproportionately affected by STIs and VH. STIs are more common among key populations in many settings, and the World Health Organization (WHO) advocates for interventions to address STIs, including strengthening surveillance and screening to be targeted to groups at higher risk [1, 2]. In high‐income countries, people who inject drugs are estimated to be involved in over 80% of ongoing hepatitis C virus (HCV) transmission [3, 4, 5], including in prisons [6]. In low‐ and middle‐income countries, transmission among key populations also contributes to hepatitis B virus (HBV) and HCV epidemics [7, 8]. Many of the structural barriers to service use faced by key populations affect their access to HIV, STI and VH services in similar ways, and the same risk behaviours, such as condomless sex and unsafe injection practices, increase the likelihood of all these infections [9].

To address HIV/STI/VH among key populations, the WHO recommends an evidence‐based package of interventions at multiple levels [9]. These include enabling interventions—such as removing punitive laws, policies and practices, reducing stigma and discrimination, community empowerment and addressing violence—which address structural barriers to health service access; health interventions—such as needle syringe programmes, condoms and lubricants, HBV vaccination, and HIV pre‐ and post‐exposure prophylaxis—that have a direct impact on HIV/STI/VH; interventions for broader health conditions, such as mental health and contraception, and supportive interventions—such as health education and demand creation—which support the delivery of health sector interventions [9]. Psychosocial interventions, such as counselling for risk behaviour change, are widely used in key population programmes which aim to reduce HIV/STI/VH transmission. While there are a range of different psychosocial interventions that may affect HIV/STI/VH prevention, in this review, we focus on counselling behavioural interventions, defined as interventions where information is exchanged and support is provided so that an individual can make decisions, design a plan and take action to reduce their risk by changing their behaviours. These interventions involve a two‐way communication between the client and the counsellor, and can be delivered through diverse facilitators (peer, health worker), frequency/duration (one session, multiple sessions) and modalities (in‐person, online). Such interventions have been shown to reduce STI risk among general populations [10]. Previous systematic reviews among key populations are limited; one review of psychosocial interventions broadly suggested that they may reduce HIV/VH/STI risk behaviours among people who inject drugs [11]. However, the impact of counselling behavioural interventions across key populations and particularly on HIV/STI/VH acquisition has not previously been synthesized.

In 2019, WHO began the process of preparing updated consolidated guidelines on HIV/STI/VH prevention, diagnosis, treatment and care for key populations. Prior WHO key population guidelines published in 2014 [12] and 2016 [13] had included recommendations related to behavioural interventions, but these were based on low‐quality of evidence, and some were difficult to interpret due to a lack of standard comparators. They also covered a wide range of potential intervention approaches, from voluntary HIV counselling and testing to peer‐based and community‐based approaches to condom promotion. These recommendations were thus identified as in need of an update. To synthesize the scientific evidence on counselling behavioural interventions with key populations, we conducted a systematic review and meta‐analysis of the effect of these interventions on HIV/STI/VH incidence, as well as complementary data on values and preferences and costs.

## 2 METHODS

Following PRISMA guidelines [14], we conducted a systematic review and meta‐analysis of the evidence in three related areas: effectiveness of the intervention, values and preferences of clients and health workers related to the intervention, and costs/cost‐effectiveness of the intervention. These are all required by the WHO Handbook for Guideline Development [15] for the guideline decision‐making process. Articles could be included in multiple parts of the review if they provided data meeting the inclusion criteria for each. The full study protocol was not registered but was submitted to WHO before the review was conducted and is available from the authors upon request.

We focused this review on counselling behavioural interventions, as defined above. We excluded studies which provided only a one‐way provision of information or education, and studies which focused on contingency management. We also excluded studies that only included counselling as part of standard HIV testing services, although we included studies that provided enhanced counselling as part of HIV testing services.

### 2.1 Effectiveness review

The effectiveness review was designed according to the Population, Intervention, Comparator, Outcome (PICO) format as follows: Do counselling behavioural interventions reduce risk behaviours associated with transmission or acquisition of HIV, STI and VH for key populations?

**P** opulations: Sex workers, men who have sex with men, people who inject drugs, people in prisons and other closed settings, and trans and gender diverse individuals (as described in the Glossary section of the 2022 WHO consolidated guidelines on HIV, VH and STI prevention, diagnosis, treatment and care for key populations [9])

**I** nterventions: Counselling behavioural interventions

**C** omparator: No intervention or a different intervention, including a different kind of counselling intervention

**O** utcome:

Primary review outcomes:

1. HBV incidence
2. HCV incidence
3. HIV incidence
4. STI incidence (e.g. syphilis)

Secondary review outcomes:

1. Unprotected sex (e.g. condomless sex, sex without lubricant, sex without HIV pre‐exposure prophylaxis [PrEP])
2. Needle/syringe sharing
3. Mortality

Secondary review outcomes were assessed only for studies that also included a primary review outcome of interest.

The inclusion criteria for this review were as follows:

1. Study design: randomized controlled trials (RCTs) that compared the intervention versus the comparison
2. Measured one or more of the outcomes of interest
3. Published in a peer‐reviewed journal from 1 January 2010 to 31 December 2022.

No restrictions were placed based on the location of the intervention or language of the publication.

We searched four databases (CINAHL, PsycINFO, PubMed and EMBASE) for relevant peer‐reviewed publications. Search terms covered terms for key populations, infections (HIV, VH, STIs) and counselling behavioural interventions. The full search strategy is available in Appendix S1. We ran this search first on 1 March 2021, for the purposes of the WHO guidelines, and then updated the search through 31 December 2022 for the purposes of presenting the most updated findings in this manuscript. The online database search was complemented in several ways. First, in 2019, we had run a search for behavioural interventions for key populations more broadly as part of an original scoping review. Articles identified through this prior search were included in the review. Second, we hand‐searched the references of included articles. Third, we contacted experts in the field (including WHO guideline development group members) to identify additional articles we may have missed. The cut‐off date of 2010 was selected *a priori* to focus on a relatively recent evidence base, as the context surrounding many key population programmes has changed substantially in recent decades.

Titles, abstracts, citation information and descriptor terms of citations identified through the search strategy were screened for initial inclusion. Full‐text articles were obtained of all selected abstracts and two independent reviewers assessed all full‐text articles for eligibility to determine the final study selection. Differences were resolved through consensus. Data were extracted independently by two reviewers using standardized data extraction forms in Excel. Differences in data extraction were resolved through consensus and referral to a senior study team member when necessary.

The following information was gathered from each included study in the effectiveness review:

- citation information (author, year, title, journal, language of article)
- location (country, urban/rural, World Bank income classification, WHO region)
- key population (sex workers, men who have sex with men, people who inject drugs, people in prisons and other closed settings, and trans and gender diverse individuals) and description (gender, age, etc.)
- sample size (*n*)
- study design (including follow‐up periods and loss to follow‐up)
- intervention description (including who delivered intervention, where intervention was provided and length/frequency of the intervention)
- comparator description
- study outcomes (per the PICO question above) (analytic approach, outcome measures/definitions, intervention vs. comparison group, number and percentage or effect sizes with confidence intervals or significance levels, conclusions, limitations)

The risk of bias was assessed using the Cochrane Collaboration's tool for RCTs [16]. Methodological components were assessed and classified as high or low risk of bias.

Data were analysed according to coding categories and outcomes. Where multiple studies reported the same outcome, we conducted a meta‐analysis using random effects models to calculate pooled risk ratios using the software program Comprehensive Meta‐Analysis. When a study included multiple comparison arms, we selected for meta‐analysis the most intensive/highest dose of counselling compared with the least intensive/lowest dose. We were unable to use funnel plots to assess bias, as we did not have the recommended number of at least 10 included studies [17]. Following the GRADE (Grading of Recommendations, Assessment, Development, and Evaluations) system [18], findings were summarized in GRADE Evidence Profile tables using GRADEPro software.

### 2.2 Values and preferences review

The same search process was used to identify studies for inclusion in the values and preferences review, which included studies presenting primary data examining the values and preferences of potential beneficiaries, communities, providers and stakeholders for counselling behavioural interventions for key populations. Values and preferences did not need to be the primary focus of included studies as long as such data were presented. These studies could be qualitative or quantitative in nature, but had to present primary data—think pieces and review articles were excluded. Values and preferences literature was summarized qualitatively and organized by study design, location and population.

### 2.3 Cost and resource needs

The same search process was used to identify studies for inclusion in the cost review, which included studies presenting primary data comparing costing, cost‐effectiveness, cost‐utility, or cost–benefit of counselling behavioural interventions for key populations. We organized cost literature into four categories (health sector costs, other sector costs, patient/family costs and productivity impacts) and within each category present it by study design, location and population.

Findings are presented separated by effectiveness, values and preferences, and cost reviews.

### 2.4 Role of the funding source

The funder of the study had no role in study design, data collection, data analysis, data interpretation or writing of the report.

## 3 RESULTS

**Figure 1:**
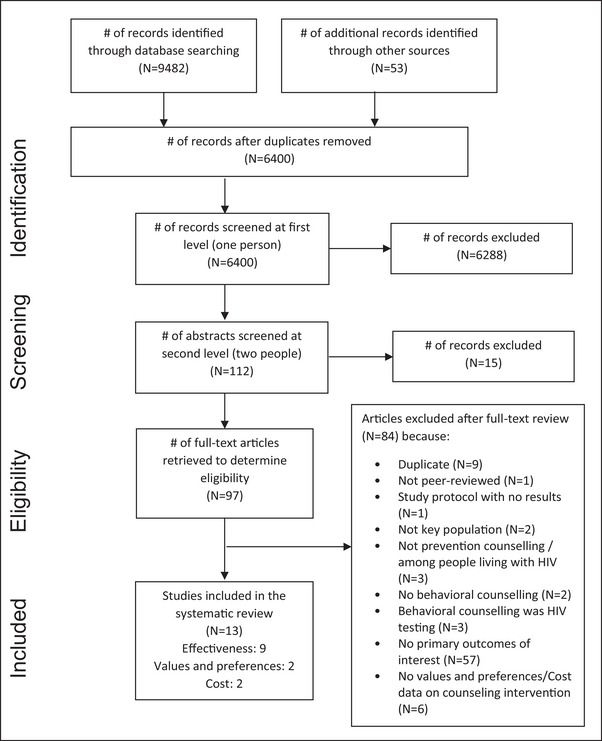
Flow chart showing the inclusion of articles across the stages of the systematic review.

We screened 6400 unique citations and identified nine articles meeting the inclusion criteria for the effectiveness review, two for values and preferences, and two for costs. These studies were all identified in the original search in 2021 that formed the basis of the WHO guidelines process; no new studies were identified for inclusion when we updated the search. Figure 1 presents the flow chart showing the inclusion of articles in the systematic review. Articles which were excluded after full‐text review for multiple reasons were counted according to their primary reason for exclusion.

### 3.1 Effectiveness review

Overall, nine studies met the inclusion criteria for the effectiveness review [19, 20, 21, 22, 23, 24, 25, 26, 27]. Table 1 provides a description of these studies. All focused on HIV/STIs; one also covered HCV. Five were conducted in the United States, while one each was conducted in Kazakhstan, China, Kenya and Mexico. Each of the five key populations was included in at least one study. Two studies were conducted with adolescent or young adult key populations—both among African Americans in the United States. One was a multi‐session intervention for 13‐ to 17‐year‐old young women being released from juvenile detention centres, while the other offered comprehensive PrEP counselling to 16‐ to 25‐year‐old men who have sex with men.

**Table 1:** Description of studies included in the effectiveness review, organized by key population

Study, country and key population	Disease focus	Intervention	Number of sessions, modality and facilitator	Comparison group	Sample size	Outcomes
Hao 2012 [25] China Men who have sex with men	HIV/STIs	Enhanced HIV voluntary counselling and testing (6‐min video, enhanced post‐test counselling, reminder for safer sex, action plan) Objective: unprotected anal intercourse reduction	Single In‐person Health worker/trained counsellor	Standard HIV voluntary counselling and testing	295	STI incidence (multiple) Unprotected sex
Desrosiers 2018 [19] USA Men who have sex with men	HIV/STIs	Personalized comprehensive PrEP counselling (needs assessments, problem‐solving for perceived barriers) Objective: PrEP initiation	Multiple In‐person Peer	PrEP education and sexual risk reduction counselling	50	HIV incidence Unprotected sex
Eaton 2018 [21] USA Men who have sex with men /trans and gender diverse individuals	HIV/STIs	Single‐session counselling intervention Objective: STI prevention and sexual risk reduction	Single In‐person Health worker/trained counsellor	Attention control on HIV/STI risk reduction	600	STI incidence (multiple) Unprotected sex
DiClemente 2014 [20] USA People in prisons and other closed settings	HIV/STIs	Multi‐session individual counselling and skills development intervention Objective: STI incidence reduction, HIV preventive behaviour improvement, psychosocial outcome enhancement	Single In‐person Health worker/trained counsellor	Usual care, including weekly group‐based HIV/STI information	188	STI incidence (multiple) Unprotected sex
El‐Bassel 2011 [24] USA People who inject drugs	HIV/STIs	Couples‐based and individual‐based counselling intervention Objective: HIV risk reduction (dual injection drug use and risky sexual practices)	Multiple In‐person Health worker/trained counsellor	Individual‐only counselling intervention and couple‐only attention control	564 (282 couples)	HIV incidence STI incidence Unprotected sex Needle/syringe sharing
El‐Bassel 2014‐effects [23] Kazakhstan People who inject drugs	HIV/STIs/VH	Group/couples‐based counselling intervention Objective: HIV/HCV/STI prevention (dual injection drug use and risky sexual practices)	Multiple In‐person Health worker/trained counsellor	Attention control on diet, physical activity and so on	600 (300 couples)	HIV incidence HCV incidence STI incidence (multiple) Unprotected sex Needle/syringe sharing
El‐Bassel 2014‐efficacy [22] USA People who inject drugs	HIV/STIs	1: Group‐based multimedia counselling intervention (psychoeducational skills building) 2: Group‐based traditional counselling Objective: HIV/STI prevention (sexual risk reduction)	Multiple In‐person Health worker/trained counsellor	Attention control on diet, physical activity and so on	306	HIV incidence STI incidence (multiple) Unprotected sex
Strathdee 2013 [27] Mexico People who inject drugs/sex workers	HIV/STIs	1: Interactive injection and sexual risk reduction intervention (including video, motivational interviewing, role‐play) 2. Interactive injection risk reduction, didactic sexual risk intervention 3: Didactic injection risk reduction, interactive sexual risk intervention Objective: safe sex and needle‐sharing reduction	Single In‐person Health worker/trained counsellor	Didactic injection and sexual risk information session	584	HIV/STI incidence (multiple)
L'Engle 2014 [26] Kenya Sex workers	HIV/STIs	6 session motivational interviewing counselling intervention Objective: alcohol use reduction	Multiple In‐person Health worker/trained counsellor	Attention control on nutrition	818	HIV incidence STI incidence (multiple) Unprotected sex

Abbreviations: HCV, hepatitis C virus; HIV, human immunodeficiency virus; PrEP, pre‐exposure prophylaxis; STI, sexually transmitted infection; VH, viral hepatitis.

Note: Risk of bias assessments are included in Appendix S2. Most effectiveness studies were rated as having some concerns according to the Cochrane risk of bias tool. This was generally due to lack of blinding (because blinding was impossible due to the nature of the interventions, although many of the outcomes should not be affected by blinding); (2) limited reporting of fidelity to the interventions (though there was no reason to doubt fidelity); and (3) participant attrition (which varied across studies and was judged to be reasonable, particularly given the challenges of retaining key populations in research studies).

Table 1 also provides a description of the counselling behavioural interventions in the included studies. Most of the interventions were brief, in‐person counselling sessions provided by a health worker. All sessions were conducted in person (face‐to‐face). Only one intervention in the United States was led by peers; all other interventions were led by health workers/trained counsellors. Four interventions were completed in a single session, while five were completed in multiple sessions. The actual content of interventions varied substantially, although all were judged to meet our definition of counselling behavioural interventions. Intervention content ranged from a focus on PrEP or enhanced voluntary counselling and testing to motivational interviewing, role‐plays, videos, or skills development components. Two were couples‐based interventions with people who inject drugs, while the rest were focused on individual behaviour change and conducted either one‐on‐one with individuals or in a group setting.

**Figure 2:**
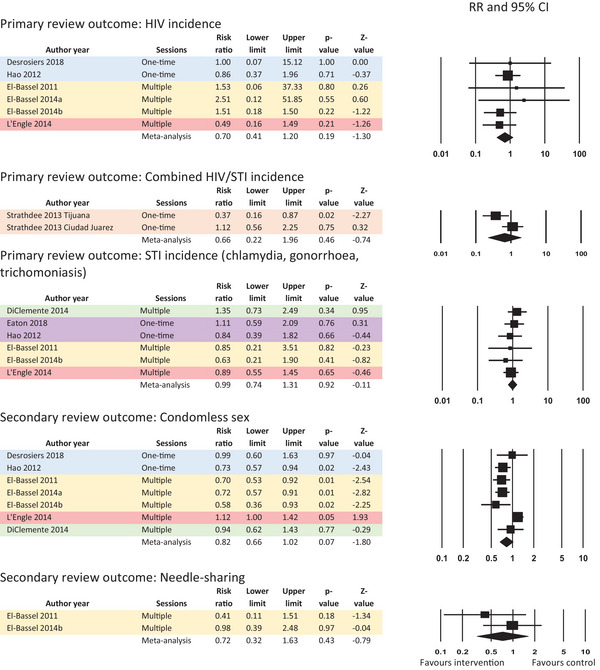
Meta‐analysis results for effectiveness review outcomes, colour‐coded by population.

The studies measured all review outcomes of interest except HBV and mortality. Meta‐analytic results are presented in Figure 2. The complete GRADE evidence profile for these studies is presented in Appendix S3. Outcomes ranged from moderate to very low certainty.

Moderate certainty evidence from six RCTs among 1280 people who inject drugs, sex workers, men who have sex with men and trans and gender diverse individuals in the United States, China, Kazakhstan and Kenya showed no impact on HIV incidence in the meta‐analysis (combined risk ratio [RR]: 0.70, 95% CI: 0.41–1.20), with no statistically significant heterogeneity (Q = 1.92, *p* = 0.86, I‐squared = 0.00).

Low certainty evidence from one RCT among female sex workers who inject drugs in Mexico showed no impact on HIV/STI incidence when findings were meta‐analysed across study sites (RR: 0.66; 95% CI: 0.22–1.96). For this analysis, we compared the most intensive counselling behavioural intervention (an interactive intervention focused on both injection and sexual risk reduction, which included a video, motivational interviewing and role‐play) versus the least intensive comparison (didactic intervention). This meta‐analysis did show statistically significant heterogeneity across the two sites (Q = 3.86, *p* = 0.05, I‐squared = 74.12) not clearly explainable by subgroup analyses or other reasons. While the authors noted that sex workers who inject drugs in Ciudad Juarez “tended to engage in higher risk sexual behaviours than their counterparts in Tijuana” [27], the original trial protocol did not plan for disaggregated analyses by study site.

Moderate evidence from six RCTs among 3783 participants from all five key populations in the United States, China, Kazakhstan and Kenya showed no impact on STI incidence in the meta‐analysis (RR: 0.99; 95% CI: 0.74–1.31), with no statistically significant heterogeneity (Q = 2.12, *p* = 0.83, I‐squared = 0.00).

Moderate certainty evidence from one RCT among 139 people who inject drugs in Kazakhstan showed no impact on HCV incidence when calculated as an unadjusted RR directly (RR: 0.45; 95% CI: 0.16–1.27), but a statistically significant reduction in HCV incidence when presented by the study authors as a rate ratio adjusting for a baseline measure of unsafe injection in the past 90 days (rate ratio: 0.31, 95% CI: 0.10–0.90).

Very low certainty evidence from seven RCTs among 1811 people in prisons and other closed settings, men who have sex with men, people who inject drugs and sex workers in the United States, China, Kazakhstan and Kenya showed no impact on the secondary review outcome of unprotected sex in meta‐analysis (using various measures of condomless sex; RR: 0.82, 95% CI: 0.66–1.02). This meta‐analysis showed statistically significant heterogeneity (Q = 22.06, *p* = 0.001, I‐squared = 72.75) which was not clearly explainable by subgroup analyses or other reasons.

Low certainty evidence from two RCTs among 564 people who inject drugs in the United States and Kazakhstan showed no impact on the secondary review outcome of needle/syringe sharing in the meta‐analysis (RR: 0.72; 95% CI: 0.32–1.63), with no statistically significant heterogeneity (Q = 1.14, *p* = 0.29, I‐squared = 11.91).

### 3.2 Values and preferences review

Two studies were included in the values and preferences review [28, 29]. Both were conducted in the United States, and both focused on counselling associated with HIV‐related topics: PrEP and HIV self‐testing. One was among men who have sex with men and one was among trans and gender‐diverse youth. Table 2 provides descriptive data for these studies, while Table 3 presents key findings. Both studies found overall high rates of satisfaction with the interventions: a two‐session motivational interviewing intervention and a video‐based counselling session.

**Table 2:** Descriptions of values and preferences studies

Study	Location	Population	Counselling behavioural intervention	Methods	Sample size
Moitra 2019 [28]	USA: Rhode Island	Men who have sex with men	Two‐session HIV PrEP motivational interviewing intervention (administered in person and by telephone through an STI clinic)	Pilot study	*N* = 19
Stephenson 2019 [29]	USA (online)	Trans and gender‐diverse individuals: binary and non‐binary youth	Project Moxie: video‐based counselling in conjunction with home‐based HIV self‐testing	Feasibility study	*N* = 201

Abbreviations: HIV, human immunodeficiency virus; PrEP, pre‐exposure prophylaxis; STI, sexually transmitted infection.

**Table 3:** Key findings of values and preferences studies

Study	Key values and preferences findings
Moitra 2019 [28]	Men who have sex with men in the United States were highly satisfied with the motivational interviewing counselling intervention and its components mean score ranged from 6.6 to 7.0 on a Likert scale ranging from 0 to 7 (with 7 best) Open‐ended feedback was generally positive especially when staff “made me feel very comfortable” One participant noted that the second (phone) session was “probably not necessary… as I was committed to taking PrEP and didn't really need a second push, but I can see it being helpful for many people.”
Stephenson 2019 [29]	Trans and gender‐diverse youth in the United States had an overall high satisfaction (98%) with video‐based counselling in conjunction with home‐based HIV self‐testing. Found their counsellors friendly (98–100%), knowledgeable (100%), experienced (87–100%) and professional (97–100%) Few reported issues with the video‐chat software and audio/visual quality, and most found it easy to use. Participants were overwhelmingly willing to repeat the intervention session later (86–100%) and would recommend the intervention to others (68–100%).

Abbreviation: PrEP, pre‐exposure prophylaxis.

### 3.3 Cost review

Two studies were included in the cost review [30, 31]. Both focused on HIV. One was conducted with young men recently released from prison in the United States, while the other was conducted with female sex workers on the United States/Mexico border. Table 4 summarizes the findings from these studies.

**Table 4:** Descriptions of cost studies

Study	Location	Population description	Counselling intervention	Methods	Sample size (*n*)
Burgos 2010 [30]	Mexico–United States border	Sex workers (women)	Mujer Segura: (1) community mobilization, promotional media, and interpersonal communication and counselling and (2) additional structural interventions, for example financial sanctions on sex establishment owners who failed to follow the intervention	Cost‐effectiveness analysis of intervention	*N* = 889
Johnson 2011 [31]	United States: California, Mississippi, Rhode Island, Wisconsin	People in prisons and other closed settings (recently released young men aged 18–29)	Project START: prelease single‐session intervention or pre‐ and post‐release multi‐session intervention on HIV/VH/STIs	Prospective cost and threshold analysis	*N* = 522

Abbreviations: HCV, hepatitis C virus; HIV, human immunodeficiency virus; STIs, sexually transmitted infections.

One cost‐effectiveness study [30] used effectiveness data from Mujer Segura, an earlier version of the intervention included in the effectiveness review among sex workers along the United States/Mexico border [27], but which was not included in the review because it was published before 2010 [32]. For a hypothetical 1000 sex workers receiving the once‐only intervention, they calculated an incremental cost of US$78,200 per 33 HIV cases prevented and 5.7 months of quality‐adjusted life years (QALYs) gained compared to no intervention. Additional cost per QALY gained was US$183. For sex workers receiving the intervention annually, they calculated an incremental cost of $US389,000, 29 additional HIV cases prevented and 4.5 additional months of QALYs compared to the once‐only intervention. The additional cost per QALY was US$1075.

Another study from the United States [31] presented prospective costs and threshold analysis for Project START: a prelease single‐session intervention or pre‐ and post‐release multi‐session intervention on HIV/VH/STIs for young men recently released from prison. Costs per participant were $689 for the single‐session comparison intervention, and ranged from $1823 to 1836 for the Project START multi‐session intervention. From the incremental threshold analysis, the multi‐session intervention would be cost‐effective if it prevented one HIV transmission for every 753 participants compared to the single‐session intervention. These costs were judged comparable with other HIV prevention programmes.

## 4 DISCUSSION

Our systematic review found no effect of counselling behavioural interventions on the incidence of HIV, VH or STIs among key populations. Evidence for effectiveness came from nine RCTs with a range of populations and was judged of moderate certainty overall, but mostly focused on HIV and STIs rather than VH. There was also no effect on secondary review outcomes of unprotected sex and needle/syringe sharing. For values and preferences, two studies from the United States with men who have sex with men and trans and gender‐diverse individuals found that participants generally viewed specific HIV‐related counselling behavioural interventions favourably. Two cost studies with sex workers in Mexico and people in prisons and other closed settings in the United States found interventions to have reasonable costs, although resource requirements will vary by setting.

Our findings show less effectiveness on behavioural and incidence outcomes than previous reviews, which found moderate effectiveness of counselling behavioural interventions with general populations (specifically excluding some key populations) [10] and of a broader set of psychosocial interventions with people who inject drugs [11] and men who have sex with men [33]. A report presented by the WHO Director‐General at the 2022 World Health Assembly notes that, despite its potential, behavioural science theory is still underused in public health and that ineffective behaviour change techniques continue to be used [34]. While this may apply to many interventions being currently implemented in key population programmes, the interventions evaluated in this review were rigorously conducted and reflected state‐of‐the‐art counselling behavioural interventions at the time they were completed—yet, they still showed no effect on HIV/VH/STI incidence. We believe that these interventions were simply insufficient, on their own, to address the myriad structural factors shaping behaviours and HIV/VH/STI risk among key populations.

Per the WHO guideline development process [15], evidence on effectiveness, values and preferences, and cost from this review was used in combination with additional considerations of equity and acceptability and input from key populations and other stakeholders to inform WHO recommendations. After deliberation, the guideline development group concluded that it was not possible to recommend counselling behavioural interventions to reduce HIV, VH and STIs given that there was no evidence of impact on the primary and secondary review outcomes of interest. However, the group felt strongly that other forms of counselling interventions such as counselling and information sharing that do not aim to change behaviour can enable informed consent and decision‐making for starting and continuing interventions, such as HIV testing, PrEP, antiretroviral treatment, and needle and syringe programmes. Counselling may enhance relationships between providers and clients and may encourage service access. Ultimately, the group agreed upon the following good practice statement and remarks, which are included in the WHO guidelines [9]:

Good practice statement:

“When planning and implementing a response for HIV, viral hepatitis and STIs, policy‐makers and providers should be aware that counselling behavioural interventions that aim to change behaviours to reduce risks associated with these infections for key populations have not been shown to have an effect on HIV, viral hepatitis or STI incidence. Counselling and information‐sharing that is not aimed at changing behaviours, can be a key component of engagement with key populations, and when provided it should be in a non‐judgmental manner, alongside other prevention interventions and with involvement of peers.”

Remarks:

“Addressing structural and social barriers is critical to create environments which permit supportive and impactful counselling”.

Counselling interventions which promote abstinence from drug use, rehabilitation or cessation of sex work or drug use, or a so‐called cure for homosexuality or gender incongruence (for example, so‐called conversion therapy)* are not recommended, and create barriers to key population service access.

* Compulsory, or involuntary, treatment for drug dependence, so‐called conversion therapy or rehabilitation of sex workers is against human rights and medical ethics principals of consent, freedom from arbitrary arrest, access to quality health, freedom from torture and cruel, inhuman and degrading treatment.

Strengths of our review include the focus on all key populations and regions, emphasis on biomedical outcomes of HIV/VH/STI acquisition for the effectiveness review, inclusion of secondary review outcomes of risk behaviour (condom use and needle/syringe sharing) and collection of complementary data on values and preferences and cost. While we conducted a thorough search using multiple databases and search strategies, we only included peer‐reviewed articles and may have missed some valuable information from the grey literature, particularly for the values and preferences and cost reviews. We also included studies published only over a 13‐year period, and although we did not exclude studies based on language, our search strategy may have missed some non‐English language publications. While our review was comprehensive for our primary review outcomes, we only included secondary review outcomes (unprotected sex, needle/syringe sharing) if they were reported in a study that also reported a primary review outcome; therefore, we did not capture all trials that reported on the secondary review outcomes, which is a major limitation of our results on behavioural outcomes. Because the number of outcomes was limited by the GRADE system used for the WHO guideline development process, we also did not include other potential outcomes of counselling behavioural interventions, such as mental health or stigma, which are important on their own and may also be on the causal pathway to incidence outcomes. Further, our primary review outcomes of interest focused on incidence, which are often rare outcomes (particularly for HIV incidence), and studies may have been underpowered to find differences in these outcomes, particularly where these were not the primary study endpoints. Finally, our results are also limited by the availability of the existing literature. Although we found RCTs conducted among all five key populations, we had few studies for any particular group, and limited data on VH. Finally, no studies measured HBV incidence or mortality.

Acknowledging these limitations, we feel that there was sufficient evidence identified in this review that further research to measure the impact of counselling behavioural interventions on HIV, STI and VH incidence among key populations is not required. However, future research could explore the effect of different aspects of counselling interventions that do not aim to change risk behaviour but focus on building client/provider relationships, psychosocial improvements (such as decreasing internalized stigma) and mental health. Such research would be useful across a range of settings and tailored for each of the key population groups.

5

In summary, our systematic review found no effect of counselling behavioural interventions on the incidence of HIV, VH or STIs, or on secondary review outcomes of unprotected sex and needle/syringe sharing. In the context of limited funding, interventions should be prioritized that have proven effective for the prevention, diagnosis and treatment of these conditions as well as on enabling interventions to address structural barriers for key populations to access these public health services. The choice to include counselling behavioural interventions in standard and minimum packages of interventions for key populations should be made with an understanding of the potential limitations on outcomes of incidence.

## COMPETING INTERESTS

The authors have no personal or financial competing interests to declare.

## AUTHORS’ CONTRIBUTIONS

AV, NL, MBM, RB and VM conceptualized the review. AV, NL, MBM, RB, VM, CEK and PTY developed the study methods and protocols. PTY and CEK conducted the search process. PTY oversaw graduate student research assistants who completed the first steps of study screening and data abstraction. CEK and PTY conducted a full‐text article review for final study inclusion, reviewed and completed data abstraction for included articles, completed risk of bias and GRADE tables, and conducted a meta‐analysis. CEK wrote the original draft. All authors contributed to writing and editing the review, and gave their assent to submit for publication.

## FUNDING

WHO receives funding from the Bill and Melinda Gates Foundation (INV‐035239) and PEPFAR/USAID for the work on key populations.

## Supporting information


**Appendix 1**: Search Strategy

**Appendix 2**: Risk of bias assessments: Cochrane tool

**Appendix 3**: GRADE evidence profile for effectiveness review studies

## Data Availability

The data that support the findings of this review are all abstracted from peer‐reviewed articles accessible from journals online and in print.
