# Supplementary material for: Counselling behavioural interventions for HIV, STI and viral hepatitis among key populations: a systematic review of effectiveness, values and preferences, and cost studies
Source: J Int AIDS Soc. 2023 May 23;26(5):e26085. doi: 10.1002/jia2.26085 (PMC10206411; doi:10.1002/jia2.26085)
Supplement: Supplementary file 1 — Appendix 1: Search Strategy [file JIA2-26-e26085-s003.docx]

**Appendix 1: Search Strategy**

**PubMed**

**Concept 1: Key populations**

Concept 1a: SW

(Sex Workers [MeSH] OR Sex work [MeSH] OR sex work [tw] OR prostitut* [tw] OR commercial sex [tw] OR transactional sex [tw] OR SW [tw] OR FSW [tw] OR CSW [tw] OR sex trade [tw] OR trade sex [tw] OR sex industry [tw] OR entertainment worker [tw])

OR

Concept 1b: MSM

(Homosexuality, Male [Mesh] OR gay men [tw] OR gay man [tw] OR gay male [tw] OR homosexual [tw] OR MSM [tw] OR men who have sex with men [tw] OR males who have sex with males [tw] OR bisexual men [tw] OR bisexual man [tw] OR bisexual male [tw] OR gay and bisexual men [tw] OR gay and other men who have sex with men [tw])

OR

Concept 1c: PWID

(Drug Users [Mesh] OR Substance Abuse, Intravenous [Mesh] OR drug use [tw] OR drug user [tw] OR drug users [tw] OR intravenous drug user [tw] OR injecting drug user [tw] OR injection drug user [tw] OR drug abuse* [tw] OR substance use [tw] OR substance abuse* [tw] OR people who inject drugs [tw] OR people who use drugs [tw] OR IVDU [tw] OR IDU [tw] OR PWUD [tw] OR PWID [tw] OR drug usage [tw])

OR

Concept 1d: TGD

(Transgender Persons [Mesh] OR transgender [tw] OR transsexual [tw] OR transvest* [tw] OR travesti* [tw] OR trans [tw] OR koti [tw] OR hijra [tw] OR mahu [tw] OR waria [tw] OR katoey [tw] OR berdache [tw] OR muxe [tw] OR third spirit [tw] OR third spirited [tw] OR assigned male at birth [tw] OR assigned female at birth [tw] OR AMAB [tw] OR AFAB [tw] OR takataapui [tw] OR tahine [tw] OR whakawahine [tw] OR tangata ira wahine [tw] OR tangata ira tane [tw] OR irawhiti [tw] OR irahuhua [tw] OR MTF [tw] OR FTM [tw] OR transmasculine [tw] OR trans masculine [tw] OR transfeminine [tw] OR trans feminine [tw] OR people of trans experience [tw] OR non-binary [tw] OR nonbinary [tw] OR gender non-conforming [tw] OR genderqueer [tw] OR gender diverse [tw])

OR

Concept 1e: PRIS (use for PICOS 1-3 but not PICO 4a-c)

(Prison [Mesh] OR Prisoners [Mesh] OR Criminals [Mesh] OR Concentration camps [Mesh] OR incarcerat* [tw] OR prison* [tw] OR jail* [tw] OR penitentiary [tw] OR penitentiaries [tw] OR penal institution [tw] OR correctional center [tw] OR correctional centre [tw] OR correctional facility [tw] OR correctional facilities [tw] OR correctional setting [tw] OR detain* [tw] OR detention center [tw] OR detention centre [tw] OR inmate [tw] OR imprison* [tw])

OR

Concept 1f: general key pops terms

(key population [tw] OR most at risk population [tw] OR MARPS [tw] OR vulnerable population [tw])

**AND**

**Concept 2: HIV, Viral Hepatitis, STIs**

Use for all PICOs

(HIV [Mesh] OR Acquired Immunodeficiency Syndrome [Mesh] OR HIV Infections [Mesh] OR human immunodeficiency virus [tiab] OR acquired immunodeficiency syndrome [tiab] OR HIV [tiab] OR AIDS [tiab] OR HIV1 [tiab] OR HIV2 [tiab] or Hepatitis, Chronic [Mesh] OR Hepatitis, Viral, Human [Mesh] OR hepatitis b [tiab] OR HBV [tiab] OR hepatitis c [tiab] OR HCV [tiab] OR hepatitis d [tiab] OR HDV [tiab] Or Sexually transmitted diseases [Mesh] OR STI [tiab] OR STD [tiab] OR sexually transmitted infection [tiab] OR sexually transmitted disease [tiab] OR sexually transmitted disorder [tiab] OR sexually transmissible infection [tiab] OR sexually transmissible disease [tiab] OR sexually transmissible disorder [tiab] OR anogenital wart [tiab] OR bacterial vaginosis [tiab] OR candida albicans [tiab] OR candidal vaginitis [tiab] OR candidiasis[tiab] OR candidosis [tiab] OR chancroid [tiab] OR chlamydia [tiab] OR Condylomata Acuminata [tiab] OR donovanosis [tiab] OR genital disorder [tiab] OR Gardnerella [tiab] OR genital infection [tiab] OR genital ulcer [tiab] OR genital wart [tiab] OR gonorrhea [tiab] OR gonorrhoea [tiab] OR Neisseria gonorrhoeae [tiab] OR granuloma inguinale [tiab] OR herpes [tiab] OR HPV [tiab] OR human papillomavirus [tiab] OR monilia albicans [tiab] OR monilial infection [tiab] OR syphilis [tiab] OR Treponema pallidum [tiab] OR trichomonas vaginalis [tiab] OR trichomoniases [tiab] OR trichomoniasis [tiab] OR venereal disease [tiab] OR venereal disorder [tiab] OR vulvitis [tiab] OR vulvovaginitis [tiab])

**AND**

**Concept 3: Behavioral counseling**

(Behavior Therapy [Mesh] OR Cognitive Therapy [Mesh] OR behavior control [tiab] OR behaviour control [tiab] OR risk reduction [tiab] OR harm reduction [tiab] OR behavioral counseling [tiab] OR behavioural counselling [tiab] OR psychosocial intervention [tiab] OR relational intervention [tiab] OR combination behavioral [tiab] OR combination behavioural [tiab] OR cognitive behavioral [tiab] OR cognitive behavioural [tiab] OR psychoeducation [tiab] OR motivational interview [tiab] OR motivational interviewing [tiab] OR counseling [tiab] OR counseling [tiab])

**CINAHL - ABSTRACT ONLY**

**Concept 1: Key populations**

(Sex Workers OR Sex work OR sex work OR prostitut* OR commercial sex OR transactional sex OR SW OR FSW OR CSW OR sex trade OR trade sex OR sex industry OR entertainment worker) OR (Male homosexuality OR gay men OR gay man OR gay male OR homosexual OR MSM OR men who have sex with men OR males who have sex with males OR bisexual men OR bisexual man OR bisexual male OR gay and bisexual men OR gay and other men who have sex with men) OR (Drug Users OR Intravenous Substance Abuse OR drug use OR drug user OR drug users OR intravenous drug user OR injecting drug user OR injection drug user OR drug abuse OR substance use OR substance abuse OR people who inject drugs OR people who use drugs OR IVDU OR IDU OR PWUD OR PWID OR drug usage) OR (Transgender Persons OR transgender OR transsexual OR transvest* OR travesti* OR trans OR koti OR hijra OR mahu OR waria OR katoey OR berdache OR muxe OR third spirit OR third spirited OR assigned male at birth OR assigned female at birth OR AMAB OR AFAB OR takataapui OR tahine OR whakawahine OR tangata ira wahine OR tangata ira tane OR irawhiti OR irahuhua OR MTF OR FTM OR transmasculine OR trans masculine OR transfeminine OR trans feminine OR people of trans experience OR non-binary OR nonbinary OR gender non-conforming OR genderqueer OR gender diverse) OR (Prison OR Prisoners OR Criminals OR Concentration camps OR incarcerat* OR prison* OR jail* OR penitentiary OR penitentiaries OR penal institution OR correctional center OR correctional centre OR correctional facility OR correctional facilities OR correctional setting OR detain* OR detention center OR detention centre OR inmate OR imprison*) OR (key population OR most at risk population OR MARPS OR vulnerable population)

**AND**

**Concept 2: HIV, Viral Hepatitis, STIs**

(HIV OR Acquired Immunodeficiency Syndrome OR HIV Infections OR human immunodeficiency virus OR acquired immunodeficiency syndrome OR HIV OR AIDS OR HIV1 OR HIV2 OR Chronic Hepatitis OR Viral Hepatitis OR hepatitis b OR HBV OR hepatitis c OR HCV OR hepatitis d OR HDV Or Sexually transmitted diseases OR STI OR STD OR sexually transmitted infection OR sexually transmitted disease OR sexually transmitted disorder OR sexually transmissible infection OR sexually transmissible disease OR sexually transmissible disorder OR anogenital wart OR bacterial vaginosis OR candida albicans OR candidal vaginitis OR candidiasis OR candidosis OR chancroid OR chlamydia OR Condylomata Acuminata OR donovanosis OR genital disorder OR Gardnerella OR genital infection OR genital ulcer OR genital wart OR gonorrhea OR gonorrhoea OR Neisseria gonorrhoeae OR granuloma inguinale OR herpes OR HPV OR human papillomavirus OR monilia albicans OR monilial infection OR syphilis OR Treponema pallidum OR trichomonas vaginalis OR trichomoniases OR trichomoniasis OR venereal disease OR venereal disorder OR vulvitis OR vulvovaginitis)

**AND**

**Concept 3: Behavioral counseling**

(Behavior Therapy OR Cognitive Therapy OR behavior control OR behaviour control OR risk reduction OR harm reduction OR behavioral counseling OR behavioural counselling OR psychosocial intervention OR relational intervention OR combination behavioral OR combination behavioural OR cognitive behavioral OR cognitive behavioural OR psychoeducation OR motivational interview OR motivational interviewing OR counseling OR counseling)

**PsycINFO ABSTRACT ONLY**

Search strategy same as CINAHL above

**Embase TITLE/ABSTRACT/KEYWORDS ONLY**

Search strategy same as CINAHL above
