# Supplementary material for: Counselling behavioural interventions for HIV, STI and viral hepatitis among key populations: a systematic review of effectiveness, values and preferences, and cost studies
Source: J Int AIDS Soc. 2023 May 23;26(5):e26085. doi: 10.1002/jia2.26085 (PMC10206411; doi:10.1002/jia2.26085)
Supplement: Supplementary file 2 — Appendix 2: Risk of bias assessments: Cochrane tool [file JIA2-26-e26085-s001.docx]

**Appendix 2. Risk of bias assessments: Cochrane tool**

| Cochrane Risk of Bias Tool (for RCTs) | | | | | | | |
| --- | --- | --- | --- | --- | --- | --- | --- |
| Risk of bias | Risk of bias arising from the randomization process | Risk of bias due to deviations from the intended interventions | | Risk of bias due to missing outcome data | Risk of bias in measurement of the outcome | Risk of bias in selection of the reported result | Overall risk of bias judgment |
| Author Year |  | Effect of assignment to intervention | Effect of adhering to intervention |  |  |  |  |
| Desrosiers et al., 2018 | Some concerns ^a^ | Some concerns ^b^ | Low ^c^ | Low ^d^ | Low ^e^ | Low | Some concerns |
| Eaton et al., 2018 | Some concerns ^f^ | Some concerns ^b^ | Low ^c^ | Some concerns ^g^ | Low ^e^ | Low | Some concerns |
| Hao et al., 2012 | Low ^h^ | Some concerns ^i^ | Low ^c^ | Some concerns ^j^ | Low ^e^ | Low | Some concerns |
| El-Bassel et al., 2011 | Some concerns ^f^ | Some concerns ^b^ | Low ^c^ | Some concerns ^k^ | Low ^e^ | Low | Some concerns |
| El-Bassel et al., 2014a | Low ^h^ | Some concerns ^i^ | Low ^c^ | Low ^l^ | Low ^e^ | Low | Low |
| El-Bassel et al.,  2014b | Low ^h^ | Some concerns ^i^ | Low ^c^ | Some concerns ^k^ | Low ^e^ | Low | Some concerns |
| Strathdee et al., 2013 | Low ^m^ | Some concerns ^b^ | Low ^c^ | Some concerns ^n^ | Low ^e^ | Low | Some concerns |
| L’Engle et al., 2014 | Low ^m^ | Some concerns ^b^ | Low ^c^ | Some concerns ^o^ | Low ^e^ | Low | Some concerns |
| DiClemente et al., 2014 | Low ^h^ | Some concerns ^b^ | Low ^c^ | Some concerns ^p^ | Low ^e^ | Low | Some concerns |

1. No information was reported about the allocation concealment. There were statistically significant differences in health insurance and PrEP desire between the two groups after randomization, but the imbalance could be the result of a small sample size (N=50).
2. Blinding was not possible due to the nature of the intervention. No information was provided about whether there were deviations from the intended intervention. However, the analysis was appropriate (modified intention-to-treat (mITT) analyses excluding participants with missing outcome data).
3. Blinding was not possible due to the nature of the intervention. However, no important deviations from the intervention were reported.
4. Only 2 participants in the control group were lost to follow-up (92% follow-up rate). So the missing data are sufficiently small that they likely made no important difference to the estimated intervention effect.
5. The method of measuring the outcome was appropriate (HIV/STI testing), and methods of measuring the outcome were comparable between groups. Lab test results could not have been influenced by participants’ knowledge of intervention received.
6. No information of concealed allocation sequence was reported. However, there were no signiﬁcant baseline differences between trial arms on any demographic variables.
7. Outcome data at the 3-, 6-, 12-month follow-up assessments were reported as a range of 82%-89% of enrolled participants in both arms. There was no evidence that the result was not biased by missing outcome data (e.g. analysis methods correcting for bias, sensitivity analyses), and missingness could depend on its true value, but we judged that missingness in the outcome was unlikely to depend on its true value because the loss to follow-up rate was similar across arms.
8. The allocation sequence was concealed and randomized. There were no signiﬁcant baseline differences between trial arms on any demographic variables.
9. Blinding was not possible due to the nature of the intervention. No information was provided about whether there were deviations from the intended intervention. However, the analysis was appropriate (intention-to-treat analyses).
10. The percentages for drop-out in the 6-month follow-up were 32.9% and 24.0%, respectively, for the two groups; this difference was statistically non-signiﬁcant. There was no evidence that the result was not biased by missing outcome data (e.g. analysis methods correcting for bias, sensitivity analyses), and missingness could depend on its true value, but we judged that missingness in the outcome was unlikely to depend on its true value because the loss to follow-up rate was similar across arms.
11. Follow-up rates were around 75%-80% and did not vary significantly among the study arms. There was no evidence that the result was not biased by missing outcome data (e.g. analysis methods correcting for bias, sensitivity analyses), and missingness could depend on its true value, but we judged that missingness in the outcome was unlikely to depend on its true value because the loss to follow-up rate was similar across arms.
12. Follow-up rates were around 90% across study arms. Multiple imputation was conducted to correct bias of missing outcome data.
13. The allocation sequence was concealed and randomized. There were no signiﬁcant baseline differences between trial arms on most demographic variables, which suggested the randomization achieved relatively balanced groups.
14. Over twelve months, only 17 participants (2.9%) did not return for at least one follow-up visit, primarily due to deaths which were unrelated to study participation (n = 10). The remaining 7 participants could not be located. Of the 567 participants who had at least one follow-up visit, an average of 12% per follow-up visit (11% in Tijuana and 13% in Ciudad Juarez) had missing data. However, testing revealed that the missing data was missing at random. There was no evidence that the result was not biased by missing outcome data (e.g. analysis methods correcting for bias, sensitivity analyses), and missingness could depend on its true value, but we judged that missingness in the outcome was unlikely to depend on its true value because the loss to follow-up rate was similar across arms.
15. Completion rates were high, with 752 participants (92%) completing at least 1 follow-up data collection visit and were comparable between groups. There was no evidence that the result was not biased by missing outcome data (e.g. analysis methods correcting for bias, sensitivity analyses), and missingness could depend on its true value, but we judged that missingness in the outcome was unlikely to depend on its true value because the loss to follow-up rate was similar across arms.
16. No significant differences were observed in attrition between the Imara intervention and the Usual Care control condition (p=.71). The overall study retention rate was 91% (n=172) at the 3-month assessment and 91% (n=171) at the 6-month assessment. There was no evidence that the result was not biased by missing outcome data (e.g. analysis methods correcting for bias, sensitivity analyses), and missingness could depend on its true value, but we judged that missingness in the outcome was unlikely to depend on its true value because the loss to follow-up rate was similar across arms.
