# Supplementary material for: Counselling behavioural interventions for HIV, STI and viral hepatitis among key populations: a systematic review of effectiveness, values and preferences, and cost studies
Source: J Int AIDS Soc. 2023 May 23;26(5):e26085. doi: 10.1002/jia2.26085 (PMC10206411; doi:10.1002/jia2.26085)
Supplement: Supplementary file 3 — Appendix 3: GRADE evidence profile for effectiveness review studies [file JIA2-26-e26085-s002.docx]

**Appendix 3. GRADE evidence profile for effectiveness review studies**

| Certainty assessment | | | | | | | № of patients | | Effect | | Certainty | Importance |
| --- | --- | --- | --- | --- | --- | --- | --- | --- | --- | --- | --- | --- |
| № of studies | **Study design** | **Risk of bias** | **Inconsistency** | **Indirectness** | **Imprecision** | **Other considerations** | **counselling behavioural interventions** | **no intervention or a different intervention** | **Relative (95% CI)** | **Absolute (95% CI)** |  |  |
| HIV incidence (follow up: range 3 months to 12 months) | | | | | | | | | | | | |
| 6 ^16,19-23,a^ | randomised trials | not serious ^b^ | not serious ^c^ | not serious | serious ^d^ | none | 9/790 (1.1%) | 10/490 (2.0%) | **RR 0.700** (0.409 to 1.197) | **6 fewer per 1,000** (from 12 fewer to 4 more) | ⨁⨁⨁◯ MODERATE ^e^ | CRITICAL |
| HIV/STI incidence (follow up: mean 12 months; assessed with: combined incidence of HIV and four STIs (syphilis, gonorrhoeae, chlamydia, and trichomonas)) | | | | | | | | | | | | |
| 1 ^24,f^ | randomised trials | not serious ^g^ | serious ^h^ | not serious | serious ^d^ | none | 26/48.11 (54.0%) | 31/47.68 (65.0%) | **RR 0.663** (0.224 to 1.960) | **219 fewer per 1,000** (from 505 fewer to 624 more) | ⨁⨁◯◯ LOW ^i^ | CRITICAL |
| STI incidence (follow up: range 6 months to 12 months; assessed with: Combined chlamydia and gonorrhoea; sometimes also including trichomoniasis) | | | | | | | | | | | | |
| 6^17,18,20-23,j^ | randomised trials | not serious ^b^ | not serious ^k^ | not serious | serious ^l^ | none | 116/1985 (5.8%) | 113/1798 (6.3%) | **RR 0.965** (0.741 to 1.308) | **2 fewer per 1,000** (from 16 fewer to 19 more) | ⨁⨁⨁◯ MODERATE | CRITICAL |
| HCV incidence (follow up: mean 12 months) | | | | | | | | | | | | |
| 1 ^20,m^ | randomised trials | not serious ^n^ | not serious ^o^ | not serious | serious ^d^ | none | 5/77 (6.5%) | 9/62 (14.5%) | **RR 0.447** (0.158 to 1.267) | **80 fewer per 1,000** (from 122 fewer to 39 more) | ⨁⨁⨁◯ MODERATE ^p^ | CRITICAL |
| HCV incidence (follow up: mean 12 months) | | | | | | | | | | | | |
| 1 ^20,m^ | randomised trials | serious ^q^ | not serious ^o^ | not serious | not serious | none | 5/67.24 | 9/45.25 | **Rate ratio 0.31** (0.10 to 0.90) | **-- per 1000 patient(s) per years**  (from -- to --) | ⨁⨁⨁◯ MODERATE ^r^ | CRITICAL |
| Unprotected sex (follow up: range 3 months to 12 months; assessed with: Condomless sex (various measures)) | | | | | | | | | | | | |
| 7 ^16,17,19-23s^ | randomised trials | serious ^t^ | serious ^u^ | not serious | serious ^l^ | none | 447/855 (52.3%) | 533/956 (55.8%) | **RR 0.821** (0.663 to 1.018) | **100 fewer per 1,000** (from 188 fewer to 10 more) | ⨁◯◯◯ VERY LOW ^v^ | CRITICAL |
| Needle/syringe sharing (follow up: mean 12 months) | | | | | | | | | | | | |
| 2 ^20,21,w^ | randomised trials | serious ^t^ | not serious ^x^ | not serious | serious ^d^ | none | 4/374 (1.1%) | 5/190 (2.6%) | **RR 0.719** (0.317 to 1.628) | **7 fewer per 1,000** (from 18 fewer to 17 more) | ⨁⨁◯◯ LOW ^y^ | CRITICAL |

**CI:** Confidence interval; **RR:** Risk ratio

#### Explanations

a. Study descriptions: RCTs among sex workers, men who have sex with men, people who inject drugs, and trans and gender diverse individuals in the United States, China, Kazakhstan and Kenya.

b. Risk of bias: Overall Cochrane risk of bias assessment across studies was "some concerns" due to risk of bias in effect of assignment to the intervention (which may lead to deviations from the intended intervention), randomization process, and missing outcome data. However, the biomedical outcome was unlikely to be influenced by knowledge of assignment to the intervention, adherence to the intervention was judged not relevant as the interventions included in this topic already range widely, and retention rates were generally around 90%. Therefore, we did not downgrade for risk of bias.

c. Inconsistency: No statistically significant heterogeneity (Q=1.916, p=0.861, I=squared = 0.000)

d. Imprecision: Downgraded because (1) 95% CI for RR includes both 1 (no effect) AND either appreciable harm (0.75) or appreciable benefit (1.25), and (2) small number of events with a wide confidence interval.

e. Number of patients: Only includes data for studies that reported this information for this outcome. Two studies that only reported effect sizes but not number of participants with the outcome were included in the meta-analysis but are not included here for the total number of patients. These were Hao et al. 2018 (n=295) and L'Engle et al. 2014 (n=818).

f. Study description: Four arm randomized trial among 584 sex workers who inject drugs in Mexico. Numbers presented here reflect the highest level counselling intervention (interactive injection and sexual risk reduction intervention including video, motivational interviewing, role play) versus the control group.

g. Risk of bias: Cochrane risk of bias assessment was judged as "some concerns" due to assignment to the intervention (which may lead to deviations from the intended intervention) and missing outcome data. However, the biomedical outcome was unlikely to be influenced by knowledge of assignment to the intervention, adherence to the intervention was judged not relevant as the interventions included in this topic already range widely, and retention rates were generally around 88-89% per session. Therefore, we did not downgrade for risk of bias.

h. Inconsistency: Statistically significant heterogeneity (Q=3.863, p=0.049, I-squared = 74.116) not clearly explainable by subgroup analyses or other reasons.

i. Number of patients: Denominators are person-years, not individual participants.

j. Study descriptions: RCTs among sex workers, men who have sex with men, people who inject drugs, people in prisons and other closed settings, and trans and gender diverse individuals in the United States, China, Kazakhstan, and Kenya.

k. Inconsistency: No statistically significant heterogeneity (Q=2.120, p=0.832, I-squared=0.000)

l. Imprecision: Downgraded once because 95% CI for RR includes both 1 (no effect) AND either appreciable harm (0.75) or appreciable benefit (1.25).

m. Study description: RCT among 300 couples (600 individuals) who inject drugs in Kazakhstan. Couples-based counselling intervention compared to attention-control group on diet and physical activity.

n. Risk of bias: Overall Cochrane assessment for risk of bias was low for this study.

o. Inconsistency: This could not be evaluated, as there is only a single study.

p. Calculated crude RR based on number of events and sample size at baseline.

q. Risk of bias: Overall Cochrane assessment for this study was low risk of bias. However, for this adjusted analysis, it was unclear whether the adjustment was part of the original protocol; it may have been a post-hoc adjustment.

r. Risk ratio: Incidence rate ratio calculated using a covariance adjustment using a baseline measure of unsafe injection in the past 90 days. The study has no published protocol, so it is unclear if this adjustment was part of the original analysis plan or was a post-hoc analysis.

s. Study descriptions: RCTs among sex workers, men who have sex with men, people who inject drugs, and people in prisons and other closed settings in the United States, China, Kazakhstan, and Kenya.

t. Risk of bias: Overall Cochrane risk of bias assessment across studies was "some concerns" due to risk of bias in effect of assignment to the intervention (which may lead to deviations from the intended intervention), randomization process, and missing outcome data. While adherence to the intervention was judged not relevant as the interventions included in this topic already range widely, retention rates were generally around 90%., the outcome was self-reported and potentially influenced by knowledge of the assignment. Therefore, we downgraded once for risk of bias.

u. Inconsistency: Statistically significant heterogeneity (Q=22.015, p=0.001, I-squared=72.746) not clearly explainable by subgroup analyses or other reasons.

v. Number of patients: Only includes data for studies that reported this information for this outcome. Four studies that only reported effect sizes but not number of participants with the outcome were included in the meta-analysis but are not included here for the total number of patients. These were Eaton et al. 2018 (n=600), El Bassel et al. 2011 (n=282), El Bassel et al. 2014a (n=306), and El Bassel et al. 2014b (n=600 couples).

w. Study descriptions: RCTs among people who inject drugs in the United States and Kazakhstan. Both couples-based counselling interventions compared to attention-control groups.

x. Inconsistency: No statistically significant heterogeneity (Q=1.135, p=0.287, I-squared=11.909)

y. Number of patients: Only includes data for studies that reported this information for this outcome. One study that only reported an effect size but not number of participants with the outcome was included in the meta-analysis but is not included here for the total number of patients. This was El Bassel et al., 2014b (n=300 couples).
